# Delayed intracranial lesions following cardiac myxoma resection in a patient with concurrent cerebral embolism: a diagnostic challenge in the absence of pathological evidence

**DOI:** 10.3389/fcvm.2026.1908269

**Published:** 2026-09-17

**Authors:** Minjie Shao, Qing Hong, Zhongming Ying, Haibo Cai

**Affiliations:** 1 Neurology Department, Wenling First People’s Hospital, Taizhou, Zhejiang, China; 2 Neurology Department, Taizhou Hospital of Chinese Traditional and Western Medicine, Taizhou, Zhejiang, China

**Keywords:** aneurysm, cardiac myxoma, case report, cerebral embolism, metastatic

## Abstract

**Background:**

Cardiac myxoma is the most common primary benign cardiac tumor and a known cause of cerebral embolism due to tumor fragment embolization. However, long-term intracranial imaging findings following surgical resection of cardiac myxoma are rarely documented.

**Case summary:**

We report a 56-year-old female with a history of intracranial aneurysm intervention, hospitalized for acute cerebral infarction. Transthoracic echocardiography (TTE) revealed a cardiac mass, which was resected and pathologically confirmed as benign cardiac myxoma. Ten months postoperatively, she developed limb convulsive seizures meeting criteria for acute symptomatic seizures. Cranial MRI with DWI showed new-onset multiple nodular hemorrhagic lesions, and PET-CT suggested myxoma recurrence with metastatic infiltration. The case was discussed at a multidisciplinary team (MDT) meeting involving neuro-oncology, neurosurgery, interventional radiology, and cardiology, At 16 months postoperatively, imaging revealed increased number and size of intracranial metastases, with worsening edema and midline shift. After full counseling, the patient and her family declined radiotherapy and biopsy. Thus, the diagnosis was based on imaging and clinical correlation, without pathological confirmation.

**Conclusion:**

This case highlights the diagnostic challenges in differentiating intracranial lesions following cardiac myxoma resection and underscores the importance of long-term cranial imaging surveillance. Even after complete tumor resection, clinicians should remain vigilant for potential myxoma-related complications.

## Introduction

Cardiac myxoma (CM) originates from multipotent endocardial mesenchymal cells, which are thought to represent embryonic remnants localized within the fossa ovalis of the interatrial septum. Its estimated incidence ranges from 0.0017% to 0.28% in the general population (1, 2). Owing to its gelatinous, friable, and inherently fragile consistency, the tumor is prone to surface fragmentation and spontaneous embolization, rendering it a well-recognized source of systemic emboli. When tumor debris dislodges into the cerebral circulation—a process frequently termed “metastasis” in the clinical literature—it typically does not infiltrate the adjacent brain parenchyma, although it may predispose the affected vessel walls to focal weakening and structural compromise (3).

Although histologically benign, CM may exhibit a biologically aggressive course characterized by distant spread. The combination of tumor embolization and local vascular invasion underpins the pathophysiological spectrum of cerebral involvement (3, 4). More specifically, the pathogenesis of CM-related ischemic stroke (CM-IS) is driven by two principal mechanisms: (i) detachment and embolization of tumor fragments, and (ii) true metastatic dissemination through hematogenous or lymphatic routes (5).

A recent study further delineated the radioclinical spectrum of cerebral manifestations associated with CM, which extends from simple embolic infarction—the most frequently encountered phenotype—to tumor emboli with vascular wall invasion that may give rise to mycotic-like aneurysms, and ultimately to frank metastatic infiltration of the brain parenchyma (6). On magnetic resonance imaging, cerebral metastases from CM characteristically present as multifocal lesions with a pronounced hemorrhagic tendency, frequently exhibiting a “popcorn-like” morphology or evidence of hemosiderin deposition on susceptibility-weighted imaging. Of note, fewer than 60 cases of cerebral metastasis from cardiac myxoma have been reported worldwide to date, underscoring the extreme rarity of this entity (7).

## Case presentation

A 56-year-old female had an intracranial aneurysm incidentally discovered during a routine health check-up. At that time, she was asymptomatic from a cardiovascular perspective, and echocardiography was not performed. The aneurysm was treated with endovascular intervention with coiling and stenting six months ago. She had been on aspirin and a statin since then with full compliance. She now presented to our emergency department with dizziness and progressive left-sided weakness, denying loss of consciousness, dysphagia, dysarthria, chest pain, palpitations, or fever.

Upon admission, initial laboratory studies revealed a markedly elevated high-sensitivity troponin I level of 2.1143 ng/mL (reference <0.0330), along with elevated D-dimer (1.37 mg/L; reference <0.50) and fibrin degradation products (3.30 mg/L; reference <2.01). In contrast, creatine kinase-MB and myoglobin remained within normal limits.

Emergency cranial CT angiography demonstrated postoperative changes at the right MCA bifurcation aneurysm with M1 segment artifact, moderate stenosis at the right posterior cerebral artery (PCA) P2 segment and left MCA M1 segment, as well as an azygos anterior cerebral artery. Electrocardiography revealed sinus rhythm with frequent atrial premature beats and nonspecific ST-T changes. Transthoracic echocardiography showed a preserved left ventricular ejection fraction of 60% and identified a 32 × 24 mm pedunculated mass arising from the left interatrial septum, accompanied by mild valvular regurgitation. This was initially reported as an indeterminate left atrial lesion warranting further clinical correlation. Pulmonary artery CT angiography (CTPA) subsequently confirmed an abnormal left atrial lesion, with features suggestive of either myxoma or thrombus, and recommended additional diagnostic evaluation (Figure 1A). Brain MRI with diffusion-weighted imaging (DWI) (Figure 2B) revealed multiple acute-to-subacute infarcts involving the right centrum semiovale, bilateral basal ganglia, right occipital lobe, left parietal lobe, and cerebellar hemisphere—a distribution pattern highly consistent with cardioembolic etiology.

**Figure 1:**
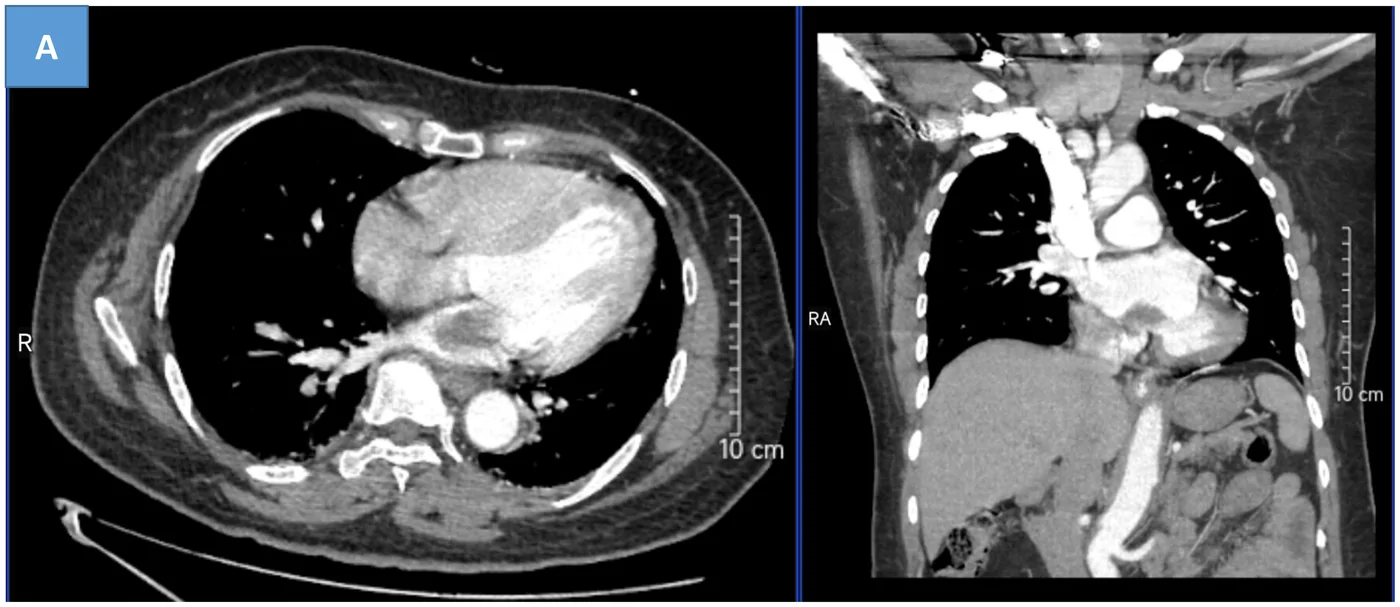
CTPA reveals a space-occupying lesion in the left atrium.

**Figure 2:**
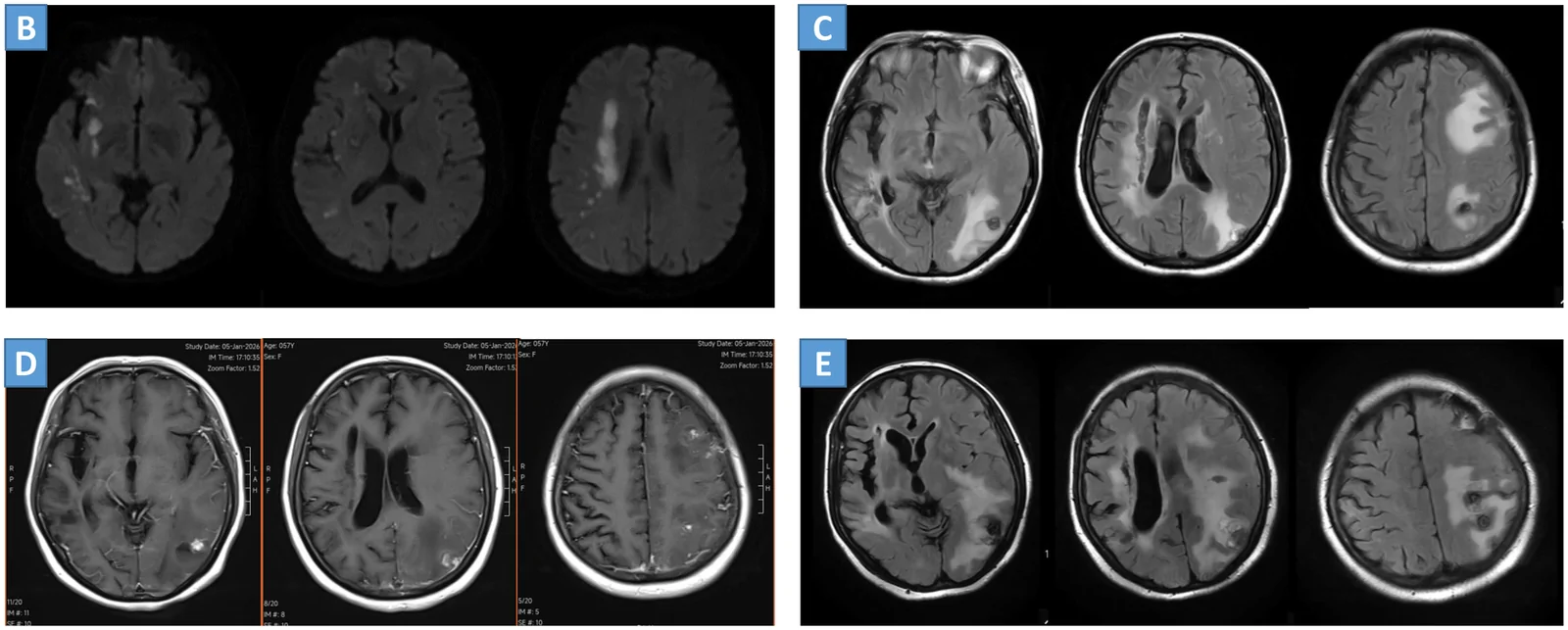
The MRI findings of this patient across different stages (2B: month 6; 2C/D: month 22; 2E: month 28).

Empiric medical therapy was promptly initiated, comprising dual antiplatelet agents (aspirin and clopidogrel), a high-intensity statin, intravenous fluid resuscitation, and adjunctive therapies aimed at enhancing collateral cerebral perfusion, along with vasodilators and neuroprotective agents. Cardiology consultation raised strong suspicion of a left atrial myxoma and advised transfer to a tertiary referral center for surgical resection following clinical stabilization.

Following a six-month period of comprehensive stroke stabilization and limb rehabilitation after the initial presentation, the patient underwent timely surgical resection of the cardiac tumor, as recommended by the cardiovascular surgery team. Pathological examination of the resected specimen demonstrated round, short spindle-shaped, or stellate cells embedded in a myxoid stroma, with abundant small-vessel hyperplasia and no evidence of nuclear atypia. These histological findings were consistent with a definitive diagnosis of left atrial myxoma.

The patient was followed up at regular intervals of one to two months. At ten months post-surgery, she developed recurrent dizziness and intermittent limb twitching, which subsequently progressed to convulsive seizures fulfilling the International League Against Epilepsy (ILAE) diagnostic criteria for acute symptomatic seizures (8) (occurring within seven days of lesion onset). Levetiracetam was accordingly initiated.

Repeat cranial MRI with DWI (Figure 2C) revealed multiple nodular lesions distributed in the left frontal, parietal, and occipital lobes. These lesions were morphologically and topographically distinct from the original infarcts. Differential diagnostic considerations at that time included calcified granulomas, parasitic infection, and metastatic disease. Subsequent non-contrast brain CT demonstrated multiple nodular hyperdensities in the corresponding regions, suggestive of hemorrhagic neoplastic lesions. Contrast-enhanced brain MRI (Figure 2D) further disclosed multiple space-occupying lesions with intralesional hemorrhage and surrounding vasogenic edema, findings highly suggestive of metastatic neoplasms, for which further diagnostic workup was strongly recommended. Positron emission tomography–computed tomography (PET-CT) was performed for comprehensive staging and revealed multiple nodular space-occupying lesions in the left frontal, parietal, and occipital regions, some of which were confluent, with the largest located in the occipital lobe. Additionally, a 1.63 × 0.70 cm patchy slightly hyperdense area was identified in the right anterior pericardium, exhibiting abnormally increased glucose metabolism, raising concern for neoplastic recurrence or pericardial involvement.

The patient was treated with corticosteroids and mannitol to alleviate cerebral edema, while levetiracetam was continued for seizure control. Upon identification of the hemorrhagic nodules, a benefit–risk assessment was urgently undertaken. Given the presence of multifocal intralesional hemorrhage and the absence of recurrent ischemic events, clopidogrel and aspirin were promptly discontinued. After stabilization of the hemorrhage, aspirin was reintroduced at a reduced maintenance dose of 100 mg daily, in order to balance the competing risks of further bleeding and in-stent thrombosis. This decision was made in close consultation with the interventional neuroradiology team.

The case was extensively discussed at a multidisciplinary team meeting involving neuro-oncology, neurosurgery, interventional radiology, and cardiology. The consensus recommendations were as follows: (i) surgical resection was considered high-risk due to the multifocal and deep-seated nature of the nodules; (ii) stereotactic radiosurgery was deferred because of the hemorrhagic characteristics of the lesions and concerns regarding radiation-induced edema and bleeding; and (iii) whole-brain radiotherapy was not advocated, given the potential neurocognitive toxicity and the unclear benefit in cases of benign metastatic myxoma.

Following the initiation of these conservative measures, the patient's clinical status remained largely stable over the ensuing months. Unfortunately, at 16 months following tumor resection (July of the current year), a follow-up brain MRI (Figure 2E) demonstrated a substantial increase in both the number and dimensions of the intracranial nodules, accompanied by marked perilesional cerebral edema and a midline shift. The patient and her family were comprehensively counseled regarding all available therapeutic alternatives and ultimately opted to continue conservative management, with close clinical monitoring and serial neuroimaging surveillance. The timeline of the patient's disease progression is shown in Table 1.

**Table 1:** Timeline of disease progression and clinical course.

**Time Point**	**Event**
**Month 0**	Intracranial aneurysm diagnosed; echocardiographic screening was not pursued; underwent interventional embolization; initiated on antiplatelet therapy
**Month 6**	Admitted for acute cerebral infarction; left atrial mass detected on echocardiography
**Month 12**	Cardiac tumor resection performed; pathology confirmed left atrial myxoma (benign)
**Month 22**	Developed recurrent dizziness, limb twitching, and seizures (acute symptomatic); MRI revealed multiple nodular hemorrhagic lesions; suspected metastasis
**Month 28**	Most recent follow-up: increased number and size of intracranial nodules, worsening edema, midline shift

## Case discussion and literature review

Cerebral metastases of cardiac myxoma demonstrate characteristic MRI features that facilitate diagnostic differentiation (9). Lesions are typically multifocal and supratentorially distributed, with a predilection for the corticomedullary junction and middle cerebral artery territories (10). A cardinal finding is intrinsic hemorrhagic propensity, manifested as T1-weighted hyperintensity and susceptibility-weighted imaging (SWI) blooming artifacts, indicative of hemosiderin deposition from recurrent microhemorrhages. T2-weighted imaging commonly reveals hyperintense lesions surrounded by vasogenic edema. Contrast-enhanced T1-weighted sequences show variable enhancement, ranging from peripheral rim to heterogeneous nodular patterns, depending on the extent of necrosis and hemorrhage. The coexistence of hemorrhagic foci of varying chronicity within a single lesion—often described as “popcorn-like"—is highly suggestive of metastatic myxoma (11).

This pattern, combined with the interval growth on follow-up imaging and PET-CT avidity, strongly favors true metastatic progression rather than recurrent embolic infarction with hemorrhagic transformation. The authors acknowledge that, although emboli do not typically infiltrate the parenchyma, rare cases of true metastatic infiltration have been reported, and the present case adds a new example to this limited body of evidence.

The reported time interval from cardiac myxoma resection to the detection of cerebral metastases varies considerably across the literature, ranging from two months to as long as eight years (12). The delayed appearance of new-onset intracranial lesions—in this case, at ten months postoperatively—warrants mechanistic consideration. Following local proliferation, the tumor cells may induce neovascularization; however, the newly formed vessels are inherently fragile and prone to increased permeability, predisposing to recurrent microhemorrhages and subsequent intratumoral bleeding (13). These cumulative hemorrhagic events may ultimately result in the development of radiologically detectable nodular lesions with hemorrhagic components. Additionally, as the patient's postoperative immune status gradually re-equilibrated, residual tumor cells may have gained local microenvironmental support—including interactions with stromal cells and inflammatory cytokines—subsequently entering a logarithmic phase of growth (14).

In the present case, several lines of evidence argue against alternative diagnoses. First, a comprehensive systemic workup was performed, including whole-body PET-CT and serum tumor marker panels, which revealed no evidence of an extracranial primary malignancy or other hypermetabolic lesions. Second, the patient presented with no constitutional symptoms (e.g., fever, night sweats, or weight loss), had no immunocompromised status, and had no prior history of malignancy. Third, serial laboratory investigations—including complete blood count, inflammatory markers (CRP, ESR), infectious disease testing (blood cultures, viral serologies, and tuberculin skin test), as well as serological screening for central nervous system demyelinating disorders—were all unremarkable. Fourth, the cardiac myxoma was pathologically confirmed after surgical resection, and the temporal association between the diagnosis of myxoma and the appearance of the intracranial lesions, combined with their imaging features on MRI and PET-CT, is most consistent with myxoma-related involvement.

This case underscores several important clinical lessons. First, Cardiac myxoma should be suspected in young patients with cryptogenic stroke, multiple infarcts, and intracranial aneurysms, even in the absence of cardiac symptoms. This case underscores the importance of echocardiographic screening in such patients, as early identification and surgical resection of the tumor may prevent further embolic events and metastatic complications. Second, the delayed appearance of intracranial metastases after seemingly curative resection suggests that the biological behavior of cardiac myxoma may be more indolent yet persistent than traditionally appreciated, warranting long-term surveillance beyond the perioperative period. Third, the potential for hemorrhagic transformation and aneurysm formation necessitates careful monitoring and individualized management strategies, particularly when antiplatelet or anticoagulant therapies are administered. Further mechanistic studies and accumulation of clinical cases are needed to better define the natural history and optimal therapeutic approach for patients with cardiac myxoma.

## Data Availability

The datasets presented in this study can be found in online repositories. The names of the repository/repositories and accession number(s) can be found in the article/Supplementary Material.

## References

[1] Marrone S Gueli IA Lo Coco R Scalia L Rizzica S Baiamonte G. Rain metastases from primary cardiac tumors: a systematic review of diagnosis, treatment, and prognosis. Cancers (Basel). (2025) 17(10):1621. [2] Aiello VD, de Campos FP. Cardiac Myxoma. Autops Case Rep. 2016, 6(2):5–7. 10.3390/cancers17101621 40427120 PMC12110033

[2] Ma K Zhao D Li X Duan H Yan C Wang S. Case report: Multiple brain metastases of atrial myxoma: Clinical experience and literature review. Front Neurol. (2023) 13:1046441. 10.3389/fneur.2022.1046441 36845225 PMC9944787

[3] Kuhn AW Lerario AM Morais ANR Iglesio RF Ramires FJA Maluf LCA. Case report: cardiac myxomas and carney complex: a case of recurrent embolic strokes and intracranial tumor growth. Front Oncol. (2025) 15:1605692. 10.3389/fonc.2025.1605692 40641912 PMC12240784

[4] Sadia H Oshikoya AF Sachdev P Fasoranti-Sowemimo OF Shawl SH Kumar K. Trial myxoma: an unusual etiology of ischemic stroke in an adult patient. Cureus. (2023) 15(6):e40599. 10.7759/cureus.40599 37469821 PMC10353846

[5] Niño AO Ramirez LAC Leal JCA Ortiz SM Cordoba LG. Brain manifestations secondary to auricular myxoma. Radiol Case Rep. (2020) 15(11):2371–4. 10.1016/j.radcr.2020.08.043 PMC7509131 32994844

[6] Reynen K. Frequency of primary tumors of the heart. Am J Cardiol. (1996) 77:107. 10.1016/S0002-9149(97)89149-7 8540447

[7] Kholoki O Al Dailaty A Atallah J Godin SL Kingma T Kim AS. Cerebral metastasis from recurrent atrial myxoma: a case report. Am J Case Rep. (2026) 27:e949724. 10.12659/AJCR.949724 41481537 PMC12801776

[8] Beghi E Carpio A Forsgren L Hesdorffer DA Malmgren K Sander JW. Recommendation for a definition of acute symptomatic seizure. Epilepsia. (2010) 51:671–5. 10.1111/j.1528-1167.2009.02285.x 19732133

[9] Solís-Gómez R Dávalos Cabral N Arrieta Limón G Hurtado Presa BA Salgado Alvear A Reyes Martínez LM. Brain metastases in a patient with antecedent of cardiac myxoma: a case report and review of literature. Rev Fac Cien Med Univ Nac Cordoba. (2024) 81(4):783–92. 10.31053/1853.0605.v81.n4.44662 PMC11905776 39670899

[10] Lao J Mu C Yu L Wu J Zhang H Guo P. Brain metastases from atrial myxoma: a delayed case report and literature review. Front Neurosci. (2026) 20:1859294. 10.3389/fnins.2026.1859294 42564132 PMC13442533

[11] Kim AH Lee J-W Lee M-K Yoon PH Kim MJ. Delayed cerebral metastases from completely resected cardiac myxoma: case report and review of literature. J Korean Soc Magn Reson Med. (2011) 15(2):165–9. 10.13104/jksmrm.2011.15.2.165

[12] Cai B Qiao M-L Miao D Liu G-Z. Cardiac myxoma and its implications for cardioembolic stroke. World J Cardiol. (2025) 17(12):112396. 10.4330/wjc.v17.i12.112396 41479996 PMC12754060

[13] Liu X Shen H Yu J Luo F Li T Li Q. Resolving the heterogeneous tumour microenvironment in cardiac myxoma through single-cell and spatial transcriptomics. Clin Transl Med. (2024) 14(2):e1581. 10.1002/ctm2.1581 38318640 PMC10844892

[14] Jiang Z Kang Q Qian H Xu Z Tong H Yang J. Revealing the crucial roles of suppressive immune microenvironment in cardiac myxoma progression. Signal Transduct Target Ther. (2024) 9(1):1–16. 10.1038/s41392-024-01912-2 39090109 PMC11294589

